# Oxcarbazepine may be an effective option for Chinese pediatric patients with self-limited focal epilepsy of neonatal/infantile onset: a retrospective cohort study

**DOI:** 10.3389/fped.2025.1509660

**Published:** 2025-04-01

**Authors:** Na Sun, Xueying Wang, Shaoping Huang, Lin Yang, Dan Li

**Affiliations:** Department of Paediatrics, The Second Affiliated Hospital of Xi’an Jiaotong University, Xi’an, China

**Keywords:** self-limited epilepsy syndromes, follow-up, outcome, oxcarbazepine, whole-exome sequencing

## Abstract

**Objective:**

The aim of this study was to evaluate the long-term follow-up data of Chinese children with self-limited focal epilepsy with neonatal/infantile onset (SeLFE) and to investigate the clinical features, genetic background and treatment outcomes of this type of epileptic syndrome.

**Methods:**

We conducted a retrospective cohort study of twenty-six children with SeLFE admitted to or followed by the Department of Pediatrics, Second Affiliated Hospital of Xi'an Jiaotong University from October 2011 to October 2021. Treatment decisions were based on the children's seizure semiology, frequency, economy, medication accessibility, allergies and other factors, and initial medications including levetiracetam, phenobarbital and oxcarbazepine. All children were followed up regularly in the outpatient clinic.

**Results:**

The 26 children, 13 male and 13 female, were followed for a mean of 54.0 (49.0, 58.5) months. Trio whole-exome sequencing (WES) revealed no pathogenic genetic abnormalities in 16 children, and known pathological genes including PRRT2, SCN2A and KCNQ2 were detected in 10 children. Thirteen children (50.0%) achieved complete seizure control after first-line monotherapy. Among the 12 patients who failed to respond to the first monotherapy, 9 patients achieved a seizure free status with oxcarbazepine, which was used as the second-line monotherapy or as add-on therapy. One patient recovered spontaneously without treatment.

**Conclusion:**

Although SeLFE is often self-limited, this study showed that complete seizure control is not always achieved with initial medication therapy. Oxcarbazepine may be an effective option for the treatment of SeLFE.

## Introduction

Epilepsy is most prevalent in infancy, with an estimated 70.1 cases per 100,000 children aged ≤2 years (1, 2). Neonatal/infantile onset self-restricted (familial) epilepsy (SeLFE) is an epilepsy syndrome with a genetic predisposition, and seizure types are predominantly focal. It is estimated that up to 72% of children with epilepsy experience focal seizures, which may or may not be followed by focal to bilateral seizures (3). SeLFE is classified into three principal categories based on the age of onset: self-limited familial neonatal epilepsy (SeLNE, 1–28 days), self-limited familial infantile epilepsy (SeLIE, 1–24 months), and self-limited familial neonatal-infantile epilepsy (SeLFNIE). SeLFE has a prevalence of 14%–18% in children with epilepsy at 2 years of age (4, 5). SeLNE has a prevalence of 5.3/100,000 (6), and SeLIE has a prevalence of 14–22/100,000 (6). Despite the favorable prognosis and the theoretical possibility of complete seizure control with first monotherapy in patients with self-limited (familial) epilepsy with neonatal/infantile onset, this is not the case in clinical practice.

Phenobarbital (PB) is one of the oldest antiseizure medications (ASMs) in clinical use, with a history dating back to 1912. It has retained a unique position within the therapeutic arsenal and remains the most widely prescribed treatment for epilepsy on a global scale. Nevertheless, a plethora of central nervous system-related adverse effects associated with the utilization of PB are progressively being acknowledged (6). These adverse effects include sedation, attention deficit hyperactivity disorder, cognitive impairment and depressed mood in children. Levetiracetam (LEV), a second-generation antiseizure medication, has been recommended for the treatment of focal seizures in infants due to its favorable profile in terms of adverse effects and medication interactions. Conversely, Oxcarbazepine (OXC) has been deemed ineffective based on the available evidence (7, 8).

Nevertheless, children with self-limited (familial) epilepsy of neonatal/infantile onset may display autosomal dominant and *de novo* pathogenic variants in genes such as KCNQ2, KCNQ3, SCN2A and PRRT2 (3). Most children with mutations in these genes are sensitive to OXC. To improve early diagnosis, enhance the efficacy of initial medication selection, and minimize the number of medications used in children, we conducted a comprehensive review of the treatment process and long-term prognosis of 26 children with self-limited (familial) epilepsy of neonatal/infantile onset who were followed up at our hospital for an extended period. This involved an in-depth exploration of their clinical characteristics and the underlying reasons for the failure of the initial medication selected.

## Methods

### Study population

In this study, children with SeLFE who were hospitalized in the Department of Paediatrics, the Second Affiliated Hospital of Xi'an Jiaotong University, from October 2011 to October 2021 were included for retrospective analysis. The inclusion criteria were as follows: (1) age of onset 0–24 months; (2) normal intelligence and motor development before and after the onset of the disease; (3) focal or focal to bilateral seizures; (4) focal epileptic discharge but no typical epileptic discharge during seizures; (5) no evidence of a lesion causing epilepsy; and (6) genetic testing results. Children diagnosed with any of the following were excluded from the study: (1) pediatric malignancies, (2) hypoglycaemia, (3) intracranial infections, (4) traumatic brain injuries, (5) hypocalcemia, and (6) symptomatic epilepsy. This study was approved by the Second Affiliated Hospital of Xi'an Jiaotong University (2023034). Informed consent was signed and provided by the patients' parents. This study was conducted in accordance with the guidelines of the Declaration of Helsinki (version 2013).

### Gene sequencing

DNA was extracted using a Blood Genome Column Medium Extraction Kit (Kangweishiji, China) Kit. Liquid hybridization was performed using an IDT xGen® Exome Research Panel v2.0 capture probe with gDNA library sequences. The whole- exome library covered all coding and some non-coding regions of 19,396 genes in the human genome, with a capture interval size of 51 Mb. High-throughput sequencing was performed with the UWI DNBSEQ-T7 sequencing platform, and the coverage of target sequence sequencing was not less than 99%. The aligned sequences were then compared with the GRCh37/hg19 reference genome using the Burrows-Wheeler Aligner (BWA) software. Subsequently, single-nucleotide polymorphisms (SNPs) and insertions/deletions (indels) were analysed using the Genome Analysis Toolkit (GATK) software. Finally, the high-quality variants were annotated in major databases (e.g., dbSNP, 1,000 Genomes, ExAC, ESP and other frequency databases, as well as OMIM, HGMD, ClinVar, etc.) using variant annotation software developed independently. Provean, SIFT, PolyPhen2-HVAR, Polyphen2-HDIV, M-Cap, Revel, Mutation Taster, and MaxEntScan shear site prediction software were utilized to analyze the harmful effects of the variants and to screen out the variants that might have a detrimental effect on the protein structure.

### Developmental assessment

As this study was concerned with the long-term outcome of SeLFE with neonatal/infantile onset, all children underwent developmental assessment at the last follow-up visit. Wechsler Preschool and Primary Scale of Intelligence (WPPSI) was used for children aged 4–6 years. The Wechsler Intelligence Scale for children (WISC) was used for patients aged 7–14 years. The Chinese version of the two instruments was revised by Gong Yaoxian of Hunan Medical University and divided into two parts: verbal and performance scales. The raw scores of the verbal and performance scales were recorded and then the verbal scale IQ (VIQ), performance scale IQ (PIQ) and full-scale IQ (FSIQ) were converted according to the age.

The criteria for selecting the first antiseizure medication were as follows: (1) diagnosed with epilepsy; (2) selection according to syndrome and seizure type (since the seizures of the children in this study were essentially partial seizures, the medication selected were OXC, LEV, PB, and topiramate (TPM); and (3) individual variations on the principle of medication selection. These variations included avoidance of antiseizure medication containing benzene rings, such as PB and carbamazepine, for allergic children; avoidance of LEV for unstable and easily agitated children; and avoidance of medication that are complicated to take and have side effects that require monitoring for parents with poorer cognition.

The initial medications and dosages used were as follows: used included LEV, 25∼60 mg/kg/day; PB, 3∼5 mg/kg/day; sodium valproate (VPA; oral solution, Sanofi Pharmaceutical Co., Ltd., 40 mg/ml), 20∼30 mg/kg/day; OXC,15∼30 mg/kg and TPM, 3∼5 mg/kg/day. If there was seizures recurrence after administration of the first medication, another medication was added or the initial medication was replaced until no more seizures occurred. Combined medication regimens included LEV + TPM, LEV + VPA, PB + OXC, PB + VPA, VPA + OXC, PB + TPM, LEV + VPA and OXC + TPM.

### Follow-up

All the children were regularly followed up in the outpatient department (once every 4–6 months if their condition was stable and once every 2–4 weeks if their condition was unstable). The following follow-up data were included in this analysis: medication, missed medication, excessive medication, medication withdrawal without permission, serum medication concentration, and adverse medication reactions. Data on seizure status during the treatment period included in the analysis were the number of seizures, duration of each seizure, comorbidities and developmental assessment.

### Data collection

We collected basic information at birth, disease information, treatment information and follow-up information from the children's electronic medical records.

### Statistical analysis

SPSS 24.0 statistical software was used for data analysis. If the continuous variables conformed to a normal distribution, they are expressed as the mean ± standard deviation. If the variables conform to non-normal distribution, they are expressed as the median (interquartile range). The categorical variables are expressed as frequencies and percentages. *p* < 0.05 was considered to indicate statistical significance.

## Results

### Baseline characteristics

Following screening on the basis of inclusion and exclusion criteria, 26 children were included in the final analysis (Table 1). Among these children, there were 13 males and 13 females. The mean follow-up was 54.0(49.0, 58.5) months. The mean age at seizure onset was 9.0 (4.8, 17.3) months. Twelve patients had a clearly relevant family history, including 9 patients with direct relatives and 3 patients with collateral relatives with SeLFE. Analysis of brain magnetic resonance imaging (MRI) results revealed no structural abnormalities. In the children's EEG examination results, abnormal EEG waves of different degrees in different regions were noted, mainly in the bilateral frontal and central areas (Table 2). Analysis of the developmental evaluation results revealed that 24 children developed normally, 1 patient had mild language and motor development delay, and 1 patient had mild motor development delay. Among the 26 children, 4 had fever triggered seizure.

**Table 1:** Classification of self-limited epilepsy syndrome (SeLFE).

Diagnosis	N (%)	Male	Onset age
Self-limited neonatal epilepsy (SeLNE)	3 (11.54)	1 (33.33)	1–13 d
Self-limited familial neonatal-infantile epilepsy (SeLFNIE)	7 (26.92)	4 (57.14)	3–13 m
Self-limited (familial) infantile epilepsy (SeLIE)	16 (61.54)	8 (50.00)	2–23 m

**Table 2:** Baseline information and EEG data.

Patient	Sex	Age at onset	Seizure type	Solitary	EEG findings
1	Female	5 m	FS-SGS	No	Bilateral frontal and central epileptic waves
2	Male	6 m	FS-SGS	Yes	Epileptic waves in bilateral frontal, central and midline areas
3	Male	6 m	FS-SGS	Yes	Epileptic waves in bilateral frontal, central and midline areas
4	Female	3 m	FS-SGS	No	Bilateral occipital, temporal lobe, right frontal lobe, central and parietal cusp slow waves
5	Male	14 m	FMS	Yes	Extensiveness *δ* waves with a few sharp waves
6	Male	23 m	FS-SGS	No	Sharp wave, sharp slow wave and spike slow wave in bilateral frontal lobes
7	Female	19 m	FS-SGS	Yes	Multiple sharp, sharp and slow waves in bilateral frontal and midline
8	Female	5 m	FS-SGS	Yes	Bilateral frontal, occipital and temporal discharges, more significant on the left side
9	Male	8 m	FS-SGS	Yes	Bilateral frontal, central and midline epileptic waves, mainly on the right side
10	Female	17 m	FS-SGS	No	Multiple sharp, sharp and slow waves in bilateral frontal and midline
11	Male	12 m	FS-SGS	No	A few epileptic waves in bilateral frontal midline
12	Male	13 m	FS-SGS	No	Full-conduction small short-range sharp slow wave
13	Male	1d	FS-SGS	No	Multifocal spikes, sharp spikes and slow waves
14	Female	2 m	FS-SGS	Yes	Frontal and central spikes during sleep, significant on the left
15	Female	15 m	FS-SGS	Yes	Epileptic waves in bilateral frontal and central regions
16	Male	17 m	FS-SGS	Yes	A few sharp waves in bilateral frontal areas
17	Female	6 m	FS-SGS	Yes	Sharp, slow and sharp waves in bilateral frontal area
18	Female	13d	FS-SGS	No	Sharp, slow and sharp waves in bilateral frontal area
19	Male	4 m	FS-SGS	Yes	Repeated small spike slow wave in bilateral frontal, central and temporal lobes
20	Male	22 m	FMS	Yes	Frontal and midline Ø wave with sharp wave, sharp and slow wave
21	Female	5d	FMS	Yes	Protruding sharp and slow waves in frontal lobe, centre and midline
22	Female	10 m	FS-SGS	Yes	Protruding sharp and slow waves in frontal lobe, centre and midline
23	Female	18 m	FS-SGS	Yes	Slightly more epileptic waves in bilateral frontal and temporal lobes
24	Male	20 m	FS-SGS	Yes	Bilateral frontal epileptic wave
25	Male	18 m	FS-SGS	Yes	Bilateral frontal epileptic wave
26	Female	5 m	FS-SGS	No	Bilateral frontal lobe δ wave

FS-SGS, focal seizure to secondary generalized seizure; FMS, focal motor seizure.

Among the 26 patients, 3 (11.5%) had focal motor seizures, and 23 (88.5%) had focal to bilateral seizures. Seventeen patients had isolated seizures, and 9 patients had no isolated seizures. All 26 children had at least one seizure cluster. The age at the time of the first seizure cluster ranged from 1 day to 36 months. Among them, the first seizure cluster occurred within 1 month of birth (1 day, 30 days) in 2 children (7.7%), within 6 months of birth in 9 children (34.6%), within 12 months of birth in 15 children (57.7%), and after the age of 12 months in 11 children (42.3%). At the time of the first seizure cluster, the mean number of consecutive seizures was 3.0 ± 1.10. Sixteen children (61.5%) had a second seizure cluster episode, with a mean of 3.2 ± 2.1 consecutive seizures, and 9 children (34.6%) had a third seizure cluster episode.

### Genetic testing results

All 26 children had undergone genetic testing. Pathogenic genes were absent for 16 children but present for 10 children. Among them, 3 patients were PRRT2 positive, 2 patients were SCN2A positive, 3 patients were KCNQ2 positive, 1 patient was ADA2 positive, and 1 patient was GRIN2A positive. Details of the genetic test results are shown in Table 3.

**Table 3:** Results of genetic mutations in 10 children.

Patient	Gene	Chromosome location	Protein change	cDNA variant	Inheritance	Mutation type	Reported (yes/no)
2	PRRT2	chr16:29 825024-2982502 5	p.R217fs*8 (p.Arg217fsTer8)	NM_14523 9:c.649_c.650(exon2)insC	Paternal	Frameshift	Yes (18, 19)
8	PRRT2	chr16:29 825024-2982502 5	p.R217fs*8 (p.Arg217fsTer8)	NM_14523 9: c.649_c.650(exon2)insC	Paternal	Frameshift	Yes (18, 19)
9	PRRT2	chr16seq[GRCH37]del(16)(p11.2p11.2)	901 bp	NM_001256443 loss1(exon2)(all)	*de novo*	Heterozygous microdeletion	Yes (20, 21)
13	SCN2A	chr2-166229841-g-a	p.Arg1319Gln	NM_001040143.1 c.3956G > A(exon20)	Maternal	Missense	Yes (15, 22, 23)
14	SCN2A	chr2:165310406	p.Val261Met	NM_021007.3 c.781G > A(exon7)	*de novo*	Missense	Yes (24–27)
19	KCNQ2	chr20:62103784-62103785	p.Tyr11fs*11 (p.Tyr11fs Tyr11)	NM_172107 c.32_c.33(exon1)insTA	Maternal	Frameshift	No
20	KCNQ2	chr20:62051020-62051020	p.Gly418Val	NM_172107.4 c.1253G > T(exon12)	Maternal	Missense	No
21	KCNQ2	chr20:62044879-62044879	p.Gly418Val	NM_172107.4 c.1687G > A(exon15)	Maternal	Missense	Yes (28–30)
22	ADA2	Chr22: 17181488	p.Lys511Gln	NM_001282225.2 c.1531A > C(exon10)	Maternal	Missense	No
24	GRIN2A	chr16:9858774	p.Iie876 > Thr	NM_14578 8 c.2627T > C(exon13)	Paternal	Missense	Yes (31)

Outcomes Among the 26 children, seizures ceased without treatment for one child (no pathogenic gene). Among the other 25 children, the first-line medication was VPA for 2 children (8.0%), TPM for 2 children (8.0%), OXC for 3 children (12.0%), LEV for 11 children (44.0%), and PB for 7 children (28%). The seizures of 13 children (52.0%) ceased after the first-line medication treatment, whereas 12 children (48.0%) still experienced seizures. Among the 13 children whose seizures stopped, LEV was used for 5, VPA was used for 1, TPM was used for 2, OXC was used for 2, and PB was used for 3. Among the 10 children with pathogenic genes, seizures stopped for 4 children, including 2 who were PRRT2 positive, 1 who was SCN2A positive and 1 who was KCNQ2 positive, while the other 6 patients still experienced seizures. Of the 11 children who still had seizures, 4 (36.4%) received secondary monotherapy with OXC, and 7 received combination therapy, 1 (9.1%) with OXC, 2 (18.2%) with VPA, 1 (9.1%) with TPM and 1 (9.1%) with TPM and OXC.

Of the 26 patients, one patient had untreated seizures that ceased spontaneously, and 13 (50.0%) patients exhibited complete seizure control after the first monotherapy. In total, 12 (46.2%) patients did not achieve seizure control with their first monotherapy. Four patients achieved seizure control with secondary OXC monotherapy, and 4 patients achieved seizure control with the addition of OXC to their current therapy. An additional 3.8% of patients were administered OXC as add-on medication to a two-medication combination to achieve final seizure control. Ultimately, only one patient continued to experience sporadic seizures. These results suggest that seizures were controlled in all patients who preferred oxcarbazepine monotherapy and that the majority of patients who did not prefer oxcarbazepine had their seizures controlled with the addition of oxcarbazepine.

Seizures ceased in all the children with pathogenic genes. Only one medication was used for three patients who were PRRT2 positive, including 2 patients for whom OXC was used and 1 patient for whom PB was used. Among the 2 SCN2A-positive patients, only OXC was used for 1 patient, while LEV + OXC was used for the other patient. Among the 3 KCNQ2-positive patients, only LEV was used for 1 patient, PB + OXC was used for 1 patient, and PB + TPM + OXC was used for 1 patient. LEV-VPA was used for the ADA2-positive patient, while VPA + OXC was used for the GRIN2A-positive patient. At the final follow-up, only one child (female, 6 years old, no pathogenic gene detected) still experienced seizures (1–2 times per month), and the current treatment plan was OXC (20 mg/kg) + TPM(5 mg/kg)+ lamotrigine (LTG,5 mg/kg)+ketogenic diet. Although seizures stopped for all 10 children with pathogenic genes, these children continued to take medication. Among the 25 children whose seizures stopped, 15 were still taking medication orally, including 9 who were taking 1 medication, 5 who were taking 2 medication and 1 who was taking 3 medication. Details are shown in Table 4.

**Table 4:** Medication treatment (dosage is expressed as mg/kg/day).

Patient	First medication treatment			Second medication treatment			Final follow-up				
	Age(months)	Medication, dosage	Outcome	Age (months)	Medication/dosage (mg/kg)	Outcome	Age (months)	WPPSI/WISC			Outcome
								PIQ	VIQ	FSIQ	
1	5.5	LEV, 25	Uncontrolled	6	Add TPM, 3.5	Uncontrolled. 2–4 times/m	72	116	100	109	Add OXC, 16 at 11 m. LEV, 25; TPM, 3; OXC, 16.
2	6	LEV, 25	Uncontrolled	11	No LEV, initiate LCM,	Stopped	52	86	93	88	Stopped. LCM
3	6	LEV, 45	Uncontrolled	24	Add VPA,15	Stopped	66	103	94	98	Stopped. VPA + LEV
4	4	PB, 4	Uncontrolled	6	No PB, initiate TPM, 5, uncontrolled. add OXC, 20 at 7 m. Stop.	Uncontrolled	56	103	101	103	Stopped. TPM + OXC
5	28	PB, 4	Stopped				70	105	106	106	Stopped. PB
6	25	VPA, 20	Stopped				75	100	95	98	Stopped. VPA
7	25	OXC, 23	Uncontrolled				76	84	106	93	Stopped. OXC
8	5.6	PB, 5.6	Stopped				52	127	98	114	Stopped. PB
9	10	LEV, 44	Uncontrolled	17	No LEV, initiate OXC, 30	Stopped	59	77	94	83	Stopped. OXC
10	18	OXC, 24	Stopped				72	108	115	116	Stopped. No medication
11	12	TPM, 3	Stopped				67	122	113	119	Stopped. No medication
12	13	No treatment					70	85	98	91	Stopped. No medication
13	0.03	PB, 5	Uncontrolled	2	OXC, 20	Stopped	58	88	91	87	Stopped. OXC
14	2	LEV, 35; PB, 3.75	Uncontrolled	3	Add OXC, 15	Stopped	56	84	92	86	Stopped. LEV + OXC
15	15	LEV, 30	Stopped				72	110	97	104	Stopped. No medication
16	19	LEV, 30	Stopped				73	94	114	103	Stopped. No medication
17	10	PB, 5	Stopped				102	91	94	92	Stopped. No medication
18	1	LEV, 20	Stopped				63	114	98	107	Stopped. No medication
19	3	LEV, 40	Stopped				58	111	114	114	Stopped. No medication
20	25	PB, 5 + LEV, 20	Uncontrolled	34	Add OXC, 50. No PB	Stopped	68	106	104	107	Stopped. LEV
21	1	PB, 5	Uncontrolled	3	Add TPM, 5	Uncontrolled	63	106	99	103	Stopped. Add OXC, 28 at 16 m. OXC + TPM + PB
22	10	LEV, 30	Uncontrolled	12	Add VPA, 20	Stopped	59	94	89	91	Stopped. LEV + VPA
23	19	OXC, 20	Uncontrolled	20	Add TPM, 5	Uncontrolled	60	78	70	72	Uncontrolled. Add LTG at 30 m. 1–2 times/year
24	21	VPA, 20	Uncontrolled	23	Add TPM, 5	Stopped	131	90	101	95	Stopped. VPA + TPM
25	36	LEV, 30	Stopped				93	101	101	100	Stopped. LEV
26	5	TPM, 4	Stopped				58	99	92	97	Stopped. No medication

LEV, levetiracetam; OXC, oxcarbazepine; TPM, topiramate; PB, phenobarbital; LCM, lacosamide; PB, phenobarbital; VPA, sodium valproate; LTG, lamotrigine. “Uncontrolled” indicates that there was still a seizure after treatment. “Stopped” indicates that there were no seizures after treatment. m, month(s). PIQ, performance intellectual quotient; FSIQ, full-scale intellectual quotient; VIQ, verbal intellectual quotient; WPPSI, the Wechsler Preschool and Primary Intelligence Assessment. WISC, The Wechsler Intelligence Scale for Children.

## Discussion

At present, the SeLFE-related genes reported include PRRT2, KCNQ2, KCNQ3, SCN2A, SCN8A, and GABRA6. PRRT2, KCNQ2 and SCN2A are common pathogenic genes (9). All 26 children in this group had undergone testing, of whom10 were found to harbour known pathogenic genes, including PRRT2, SCN2A and KCNQ2.

The mutation site in the PRRT2 gene in 3 patients was c.649 (exon 2), which was consistent with other reports (10, 11). The PRRT2 gene mutation has obvious phenotypic heterogeneity; heterozygous mutation of this gene often causes SeLFE, whereas homozygous mutation can cause epilepsy with intellectual disability (12). In this study, 3 children with PRRT2 gene heterozygous mutations presented with focal to bilateral tonic-clonic seizures. After medication treatment (1 patient was given lacosamide, 1 patient was given PB, and 1 patient was given OXC), none of them experienced seizure recurrence. Their seizures were completely controlled with monotherapy, in line with findings previously reported in the literature.

Mutation of the SCN2A gene has been confirmed to cause epilepsy, dyskinesia, autism spectrum disease, intellectual disorders, paroxysmal ataxia, paroxysmal hemiplegia and other neurological diseases. Epilepsy caused by mutation of the SCN2A gene manifests as benign neonatal/infant epilepsy with a good prognosis but can also manifest as Ohtahara syndrome, West syndrome, Dravet syndrome, epilepsy of infancy with migrating focal seizures, Lennox–Gastaut syndrome, genetic epilepsy with febrile seizures plus and other severe epileptic encephalopathies that cannot be classified (13, 14). In this study, 2 children with SCN2A gene mutations presented focal seizures followed by generalized seizures. For both children, the age of onset was within 3 months of birth, and these children continue to take medication (1 is taking OXC, and 1 is taking LEV + OXC) without the recurrence of seizures. Wolf et al. suggested patients with early-onset epilepsy (age of onset ≤3 months) respond relatively well to sodium channel blockers (15), which is consistent with our observations.

KCNQ2 mutations can cause epilepsy in infants with different degrees of seizure severity. In our study, 3 children with KCNQ2 gene mutations were found, 2 of whom experienced focal motor seizures (1 patient continued to take LEV, but seizures did not reoccur; 1 patient continued to take OXC, TPM and PB, but seizures did not reoccur), and 1 patient experienced focal seizures followed by generalized seizures. We also found that most KCNQ2-associated epilepsies responded well to sodium channel blockers (2), especially OXC.

Our results confirm that OXC is a valuable therapeutic option for the treatment of infants and children (<2 years of age) with partial epilepsy. An analysis of a database in the United States showed that OXC was prescribed in only 3.5% of children under 1 year of age and 14.8% of children 1–4 years of age, which is much lower than the proportion of children prescribed LEV, which was 32.7% and 58.1%, respectively (16). A study by Binyang Zhao (17) et al. found that the efficacy of LEV and OXC as a single agent in the treatment of focal infantile epilepsy was 41% and 73.5%, respectively. In our study the SeLFE-related genes included PRRT2, KCNQ2, GRIN2A, SCN2A and ADA2. These genes were generally sensitive to oxcarbazepine, which led to the failure of the first medication selection in about 50% of the children, who eventually achieved seizure freedom by switching or adding oxcarbazepine. Based on this finding, we could infer that the first selection of oxcarbazepine or carbamazepine for patients with SeLFE is expected to increase the rate of a complete seizure control and reduce the chances of comorbid medication.

This study has several limitations. First, the single-centre retrospective nature of this study may have led to inclusion bias. Second, the sample size was small, which may have led to case selection bias. Finally, the neurological, mental and physical development of the children was not recorded in detail, and the follow-up time was short, so it was not possible to analyse the long-term impact of SeLFE with these children.

It should be noted that SeLFE may exhibit a diagnostic delay. Furthermore, a choice for the incorrect initial pharmacological intervention may result in suboptimal seizure control, thereby increasing the likelihood of concomitant use of two or even three antiseizure medications. In certain cases, oxcarbazepine may be the optimal single-medication choice for SeLFE.

## Data Availability

The raw data supporting the conclusions of this article will be made available by the authors, upon reasonable request. Requests to access these datasets should be directed to Dan li, runningtortoise@163.com.
